# N6-methyladenosine regulator-mediated methylation modification patterns and immune infiltration characterization in Polycystic Ovary Syndrome (PCOS)

**DOI:** 10.1186/s13048-023-01147-9

**Published:** 2023-04-12

**Authors:** Sihan Zhou, Rui Hua, Song Quan

**Affiliations:** 1 Center for Reproductive Medicine, Department of Obstetrics and Gynecology, Nanfang Hospital, Southern Medical University, Guangzhou, China; 2 The First School of Clinical Medicine, Southern Medical University, Guangzhou, China

**Keywords:** Polycystic ovary syndrome, N6-methyladenosine, M6A regulators, Bioinformatics analysis

## Abstract

**Background:**

Polycystic ovary syndrome (PCOS) is a multisystem-related disease whose pathophysiology is still unclear. Several regulators of N6-methyladenosine (m6A) modification were confirmed to play a regulatory role in PCOS. Nonetheless, the roles of m6A regulators in PCOS are not fully demonstrated.

**Materials and methods:**

Four mRNA expression profiling microarrays were obtained from the Gene Expression Omnibus (GEO) database. Differentially expressed m6A regulators between PCOS and normal patients were identified by R software. A random forest modal and nomogram were developed to assess the relationship between m6A regulators and the occurrence risk of PCOS. A consensus clustering method was utilized to distinctly divide PCOS patients into two m6A subtypes (m6A cluster A/B). The patterns of differential expression and immune infiltration were explored between the two m6A clusters.

**Results:**

In this study, 22 significant m6A regulators were identified between healthy controls and PCOS patients. The random forest model determined three optimal m6A regulators which are related to the occurrence risk of PCOS, including YTHDF1, RBM15 and METTL14. A nomogram was established based on these genes, and its predictive reliability was validated by decision curve analysis. The consensus clustering algorithm distinctly divided PCOS cases into two m6A subtypes. The ssGSEA algorithm found that the immune infiltration was markedly enriched in m6A cluster B than in cluster A. The m6A-pattern related differentially expressed genes (DEGs) of the two m6A subtypes were demonstrated by differential expression analysis. We found that they were enriched in immune-related genes and various infection pathways. Based on the m6A-pattern related DEGs, the PCOS patients were classified into two m6A-pattern related genomic subtypes (gene clusters A and B).

**Conclusions:**

The present study provided evidence concerning the different modification patterns of m6A regulators in PCOS compared with normal patients. This study will help clarify the overall impact of m6A modification patterns and related immune infiltration on PCOS.

## Introduction

Polycystic ovary syndrome (PCOS) is a multisystem-related disease of the reproductive system characterized by the pathological accumulation of follicular immaturity and atresia, ovarian and interstitial dysplasia, hyperandrogenemia (HA), hyperinsulinemia, insulin resistance (IR), metabolic abnormalities, endocrine disorders and polycystic ovary [1, 2]. Previous studies have shown significant advances in factors likely involved in PCOS pathogenesis, including the potential roles of androgen, insulin, AMH, TGFβ, oxidative stress, pro-inflammatory cytokines, advanced glycation end products (AGEs), and so on [3, 4], among which the HA seemed to be the most vital determinant of the PCOS pathophysiology and the associated metabolic dysfunction [5]. Due to the high heterogeneity and complexity of this multifactorial disease, the molecular basis of the etiology of PCOS is not fully understood yet. Therefore, a deeper investigation of the molecular mechanisms underlying the pathogenesis of PCOS is urgently needed to facilitate the development of therapeutic modalities and improve the prognosis of patients.

N6-methyladenosine (m6A), one of the most prominent and abundant epigenetic modification forms of mRNA and lncRNA in eukaryotic cells, is a dynamic, reversible, and highly conservative process under the regulation of methyltransferases, demethylases, and binding proteins [6–8]. These regulating proteins of m6A modification are also called "writers," "erasers," and "readers" of m6A [9–11]. Increasing pieces of evidence have shown that m6A modification plays crucial roles in physiological processes and disease progressions [12], such as embryonic development [13, 14], stress response, immunity [15], cell proliferation and self-renewal ability [16], tumorigenesis and metastasis [15, 17], and so on.

Previous studies have investigated and revealed the significant role of m6A RNA methylation regulators in the occurrence and progression of PCOS [18]. However, these researches concentrated predominantly on several of the 26 m6A regulators. Therefore, the function of m6A regulators in PCOS remains to be further investigated.

Since the m6A modification might play a part in the dysregulation of various systems, which provides an orientation for investigation in PCOS. Concurrently, the development of bioinformatics analysis and public databases, such as the Gene Expression Omnibus (GEO) [19], provides means to understand the molecular pattern of m6A regulators in PCOS. In this research, we comprehensively assessed the effects of m6A regulators on the occurrence risk and subtype categorization in PCOS. We constructed a nomogram to visually display the scoring coefficient and correlation between the occurrence risk of PCOS and the three optimal m6A regulators (YTHDF1, RBM15 and METTL14). As a result, PCOS patients could receive clinical benefits from this nomogram. Additionally, we identified two m6A subtypes closely associated with different immune infiltrations. This study uncovered two different patterns of m6A regulators in the granulosa cells of PCOS, which provides a theoretical foundation for further research into m6A modification and new therapeutic strategies for PCOS.

## Materials and methods

### Data extraction and processing

The RNA sequencing (RNA-seq) data of the GSE34526, GSE80432, GSE102293, and GSE137684 datasets was downloaded by using Perl (www.perl.org/) from the GEO database (http://www.ncbi.nlm.nih.gov/geo/) after searching for all expression microarrays that matched PCOS keywords with unnecessary information removed. The expression profiles of 19 controls and 25 PCOS patients from 4 datasets were combined, and the batch normalization of the read count values was completed to preserve data consistency by the "limma" and "sva" R packages.

### Differential expression analysis of m6A RNA methylation regulators

A total of 26 m6A RNA methylation regulators were collected from previous studies, including 9 writers, 15 readers, and 2 erasers (Table 1). Differentially expressed analysis of these regulators based on the limma (Linear Models for Microarray Data) algorithm was performed between control and PCOS patients to identify the DEGs of the m6A regulators. A protein–protein interaction (PPI) analysis of the above DEGs was accomplished by the STRING website (https://string-db.org/), and the hub genes were extracted by Cytoscape's cytoHubba plug-in. The gene set variation analysis (GSVA) enrichment analysis was conducted to assess the variations in biological processes to match the biological function between control and PCOS patients.

**Table 1:** Three types of m6A modification regulators and their primary biological functions

Type	Gene symbol	Abbreviation	Function
Writers	Methyltransferase-like 3	METTL3	Catalyze the formation of the m6A residues
	Methyltransferase-like 14	METTL14	Facilitate METTL3 for RNA-binding
	Methyltransferase-like 16	METTL16	Catalyze m6A modification
	Wilms tumor 1-associated protein	WTAP	Facilitate accumulation of METTL3 and METTL14 to nuclear speckle
	Vir-like m6A methyltransferase-associated	VIRMA	Recruit the m6A complex and guide it to specific site
	Zinc finger CCCH-type containing 13	ZC3H13	Bridge WTAP to the mRNA-binding component RBM15/RBM15B
	RNA binding motif protein 15	RBM15	Bind target RNAs and recruiting the WMM complex
	RNA binding motif protein 15B	RBM15B	Bind target RNAs and recruiting the WMM complex
	Cbl proto-oncogene like 1	CBLL1	Associate component of the WMM complex with unknown function
Readers	YTH domain containing 1	YTHDC1	Bind m6A-containing mRNAs and promotes recruitment of SRSF3
	YTH domain containing 2	YTHDC2	An RNA-induced ATPase with the 3' → 5' RNA helicase activity
	YTH N6-methyladenosine RNA binding protein 1	YTHDF1	Mediate m6A-containing mRNAs targets degradation
	YTH N6-methyladenosine RNA binding protein 2	YTHDF2	Regulate mRNA stability
	YTH N6-methyladenosine RNA binding protein 3	YTHDF3	Promote protein synthesis and affect methylated mRNA decay
	Heterogeneous nuclear ribonucleoprotein C	HNRNPC	Responsible for pre-mRNA processing
	Fragile X messenger ribonucleoprotein 1	FMR1	Regulate mRNA splicing, stability, dendritic transport and postsynaptic local protein synthesis
	Leucine rich pentatricopeptide repeat containing	LRPPRC	Regulate RNA metabolism in both nuclei and mitochondria
	Heterogeneous nuclear ribonucleoprotein A2/B1	HNRNPA2B1	Promote pri-miRNAs processing
	Insulin like growth factor binding protein 1/2/3	IGFBP1/2/3	Inhibit IGF-mediated growth and developmental rates
	RNA binding motif protein X-linked	RBMX	Regulate gene transcription and pre-mRNAs splicing
	ELAV like RNA binding protein 1	ELAVL1	Improve mRNA stability
	Insulin like growth factor 2 mRNA binding protein 1	IGF2BP1	Improve mRNA stability
Erasers	Fat mass and obesity-associated protein	FTO	Catalyze the demethylation of m6A
	AlkB homolog 5	ALKBH5	Demethylate m6A RNA by oxidative demethylation

### Models comparison, selection, and establishment

Random forest (RF) and support vector machine (SVM) models were established respectively as training models to predict the prevalence of PCOS. Reverse cumulative distribution of residuals, boxplots of residuals, and receiver operating characteristic (ROC) curves were conducted to assess the accuracy of models. The m6A regulators with an importance value over 2 were considered as PCOS-specific genes.

### Establishment of the nomogram

Based on the m6A regulators with an importance value over 2, a nomogram was built with the "rms" package of R to predict the occurrence risk of PCOS, as well as to demonstrate the correlation between the occurrence risk and selected m6A regulators. The calibration curve assessed the predictive accuracy and reliability of this nomogram. The decision curve analysis (DCA) was conducted to evaluate the benefits of decisions from the nomogram. A clinical impact curve was established to assess the rationality and benefit of decisions.

### Identification of subtypes based on m6A regulators

Consensus clustering with K-means algorithms was applied to identify m6A regulator-related subtypes correlated with gene expression. The quantity and robustness of clusters were determined with a consensus clustering algorithm realized in the "ConsensuClusterPlus" R package. The principal component analysis (PCA) was used to confirm the m6A subtypes.

### Identification of DEGs between m6A subtypes

The "limma" R package was applied to identify DEGs among different m6A subtypes, named m6A-related DEGs, with the criterion of adjusted *p* < 0.01 and |logFC|≥ 1.5. GO and KEGG enrichment analysis were utilized to investigate the potential function of the DEGs between m6A-subtypes responsible for PCOS via the "clusterProfiler" package in R. Based on the expression of m6A-related DEGs, the m6A-related gene subtypes were identified.

### Establishment of the m6A gene signature

To quantify the m6A regulator patterns of PCOS, a scoring system—the m6A gene signature was constructed to evaluate the m6A regulator patterns of PCOS patients, which we termed as m6Ascore. The PCA was conducted to obtain m6Ascore of each PCOS patients. Both principal components 1 and 2 were selected to act as signature scores, and m6Ascore was acquired based on the following method: m6Ascore = ∑(PC1i + PC2i), of which PC1/2 refers to principal component 1/2 and i to the expression of m6A-related DEGs.

### Estimation of immune cells infiltration

The single sample gene set enrichment analysis (ssGSEA) was applied to assess the infiltration of immune cells among different subtypes. Immune cells include immune-enhancing cells and immune-suppressive cells. The gene expression levels of the patients were sequenced with ssGSEA to acquire an infiltration grade. Then the expression data were summarized for immunological analysis. Differential expression analysis with moderated t-tests would be utilized to assess the enrichment of immune cells and the distribution of pro-inflammatory cytokines between different subgroups, and *p* < 0.05 was recognized as a significant result.

### Statistics analysis

Linear regression analyses were applied to determine the relationship between m6A regulators. Kruskal–Wallis tests were utilized to identify a discrepancy between subtypes. All statistical analyses were carried out with two-tailed tests, and the significant value was considered *p* < 0.05. R × 64 (version 4.1.2) and packages in R were utilized to run and process data scripts, output results, and plot diagrams.

## Results

### The landscape of the m6A regulators in PCOS

**Fig. 1:**
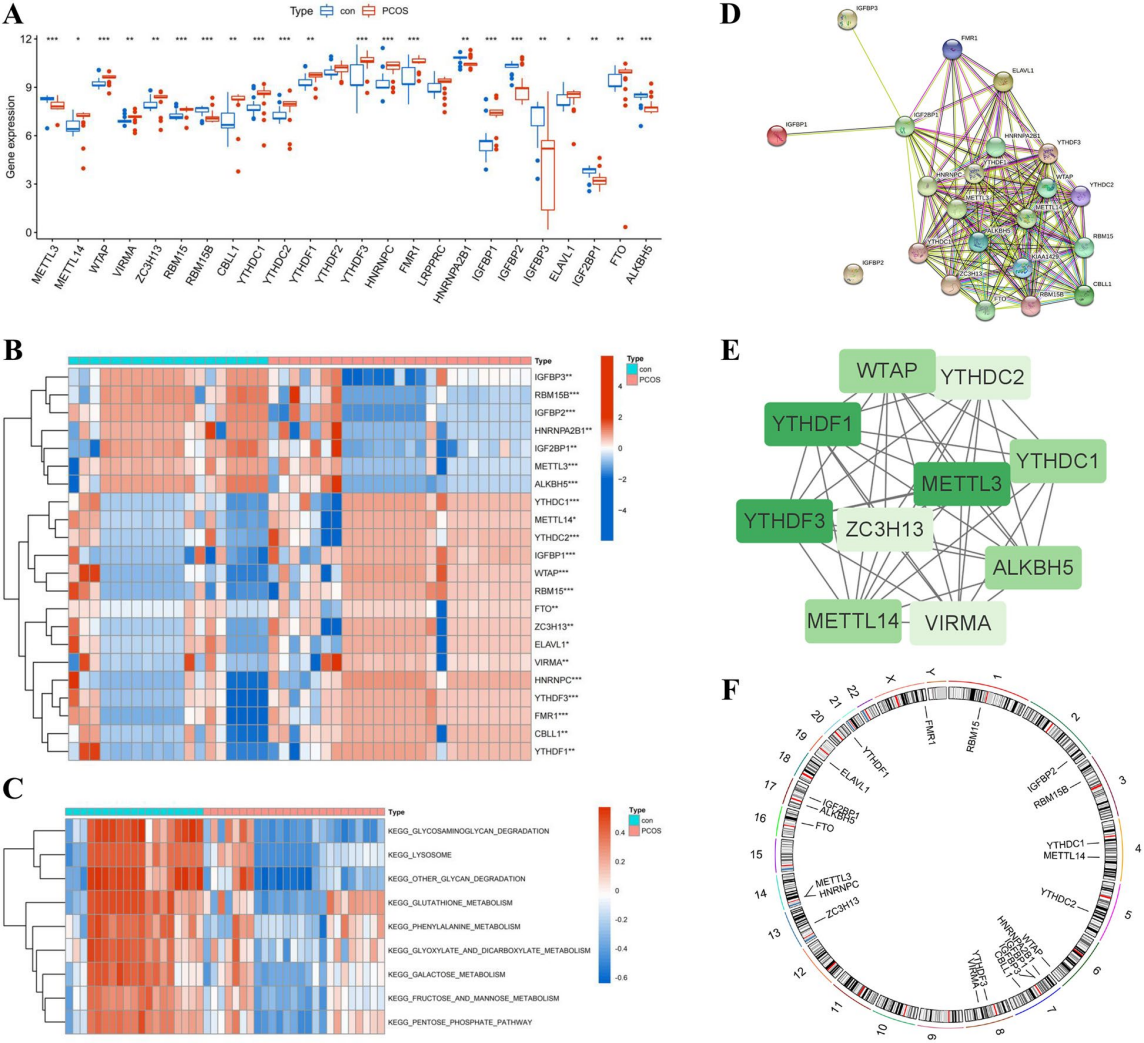
Landscape of m6A RNA methylation regulators in PCOS. **A** Differential expression analysis of m6A regulators between control and PCOS patients named “con” and “PCOS” respectively, and the gene expression has been converted by log2(counts + 1). **B** Expression heatmap of the identified 24 m6A regulators in two groups. **C** GSVA enrichment analysis between PCOS and control group. **D** PPI network analysis of the DEGs in m6A regulators. **E** Top 10 hub genes of m6A regulators in PPI network. **F** Chromosomal positions of the 22 DEGs of m6A regulators. *p < 0.05, **p < 0.01, and ***p < 0.001

Based on the selected datasets, the "limma" R package was applied to analyze differential expressions of 26 m6A regulators between control and PCOS patients. The complete expression data of 24 m6A regulators was successfully retrieved. The expression landscape of these m6A regulators was visualized in a histogram and a heatmap (Fig. 1A, B). Subsequently, 22 DEGs were observed in the differently expressed analysis of m6A regulators between control and PCOS samples. Most of the DEGs were overexpressed in PCOS compared to the control group, including METTL14, WTAP, VIRMA, ZC3H13, RBM15, CBLL1, YTHDC1, YTHDC2, YTHDF1, YTHDF3, HNRNPC, FMR1, IGFBP1, ELAVL1, and FTO, and several other DEGs such as METTL3, RBM15B, HNRNPA2B1, IGFBP2, IGFBP3, IGF2BP1, and ALKBH5 were downregulated in PCOS. The GSVA analysis demonstrated that the glyoxylate and dicarboxylate metabolism, glycosaminoglycan degradation, phenylalanine metabolism, fructose and mannose metabolism, and lysosome were obviously enriched in the control group than in PCOS patients (Fig. 1C). The PPI analysis demonstrated the interactions of 22 DEGs of m6A regulators, and Cytoscape illustrated three hub genes, METTL3, YTHDF1, and YTHDF3 (Fig. 1D, E). Additionally, the 22 DEGs of m6A regulators were mapped onto chromosomes by the "RCircos" package in R (Fig. 1F).

### Correlation analysis between m6A regulators in PCOS

**Fig. 2:**
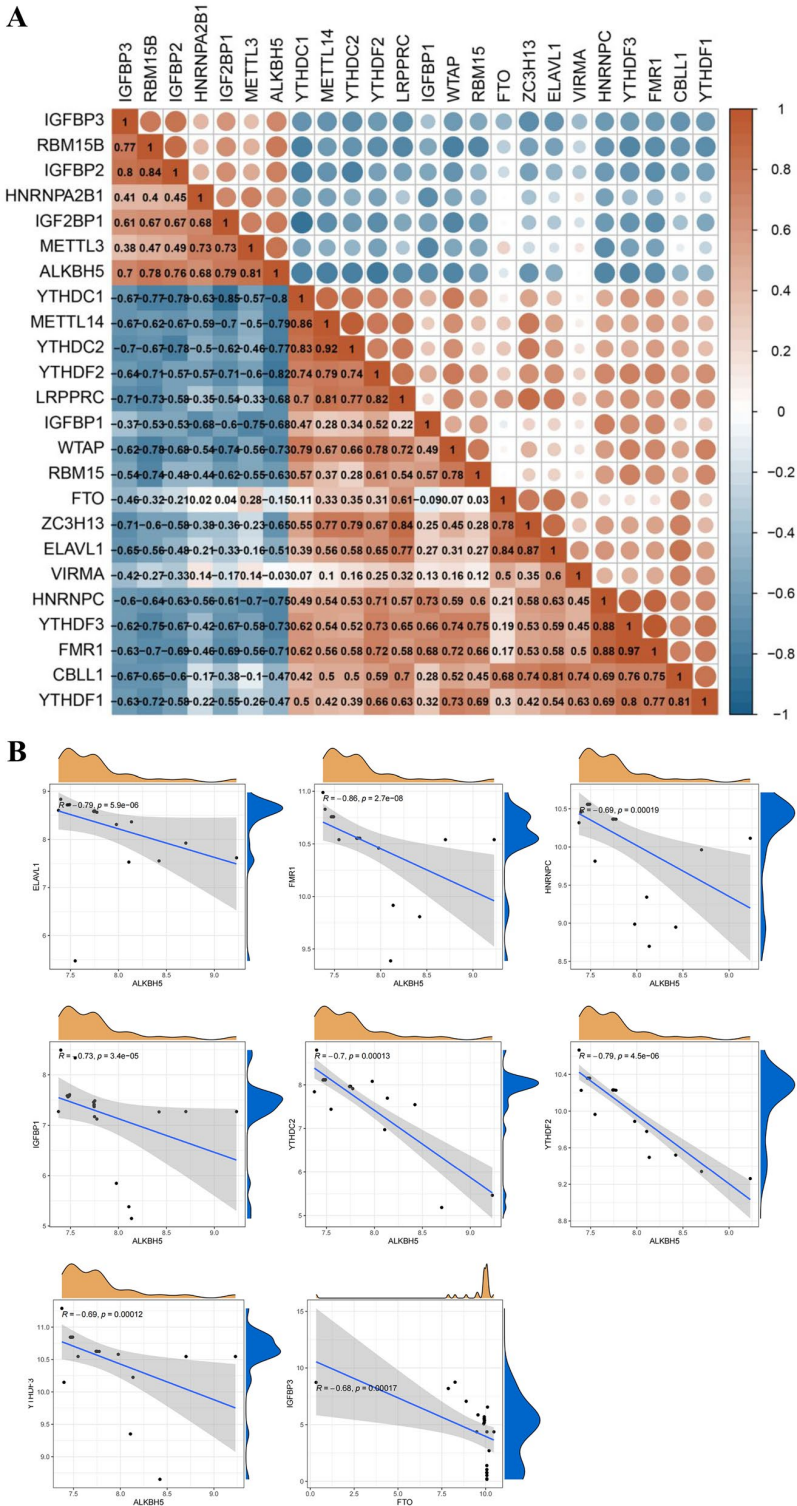
Correlation analysis of m6A regulators in PCOS. **A** Correlation heatmap of 24 m6A regulators. **B-I** Correlation between m6A readers and erasers in PCOS. Reader genes: ELAVL1, FMR1, HNRNPC, IGFBP1, YTHDC2, YTHDF2, YTHDF3, IGFBP3; eraser genes: ALKBH5 and FTO

The correlations between the m6A regulators are presented in Fig. 2A. The m6A regulators could display positive correlations, such as FMR1 and YTHDF3 (coefficient = 0.97), and negative correlations, such as YTHDC1 and IGF2BP1 (coefficient = -0.85). To investigate the possibility of co-expression between m6A regulators, we discovered a clear relationship between YTHDF3 and other regulators, with the greatest relevance for it with FMR1 as mentioned earlier, which was consistent with the PPI analysis. The correlation heatmap shows that the readers and erasers of m6A regulators in PCOS were highly related. Linear regression analyses were applied to further investigate the relationship between readers and erasers. Significant negative correlations could be observed between ELAVL1, FMR1, HNRNPC, IGFBP1, YTHDC2, YTHDF2, YTHDF3, and ALKBH5 in PCOS patients. Figure 2B-H illustrate that PCOS patients with low ALKBH5 expression levels tend to present high levels of above readers. And the expression of FTO demonstrated a negative association with IGFBP3 in PCOS (Fig. 2I).

### Evaluation of the RF model and SVM model

**Fig. 3:**
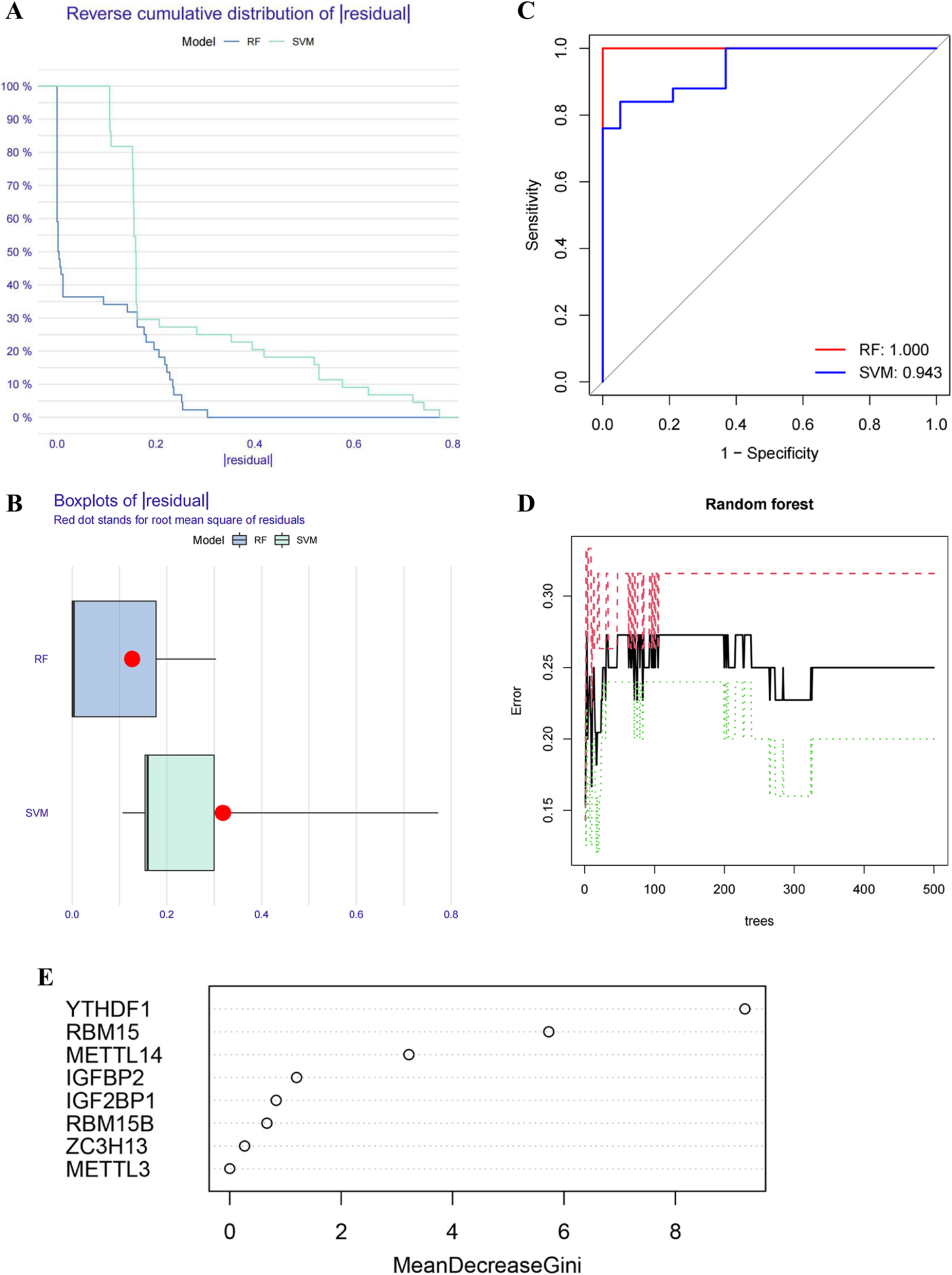
Establishment and comparison of the RF model and SVM model. **A** Reverse cumulative distribution of |residual| of RF and SVM model. **B** Boxplots of |residual| for displaying the residual distribution of RF and SVM model. **C** ROC curves demonstrated the accuracy of the RF and SVM mode. **D** The random forest model displayed the influence of the number of decision trees on the error rate. **E** The importance of the m6A regulators based on the RF model

Based on 22 DEGs of m6A regulators, the RF model and SVM model were respectively constructed to identify optimal m6A regulators in order to evaluate the occurrence risk of PCOS. According to the "reverse cumulative distribution of residuals" and "boxplots of residuals" (Fig. 3A, B), the RF model retained smaller residuals, indicating that the RF model's predictive performance has more superiority to the SVM model. Moreover, the ROC curves were established to assess the accuracy of two models, and the AUC value also demonstrated the better performance of the RF model (Fig. 3C). Consequently, the RF model was applied to screen for the signature genes of PCOS. The RF model displayed the relationship between the error and the number of decision trees to find the point with the smallest cross-validation error for choosing the optimal number of decision trees (Fig. 3D). Based on the RF model, the DEGs of m6A regulators were given their respective gene importance, and gene importance rank demonstrated that YTHDF1, RBM15, and METTL14 had priorities in the model (Fig. 3E).

### Evaluation of a predictive nomogram

**Fig. 4:**
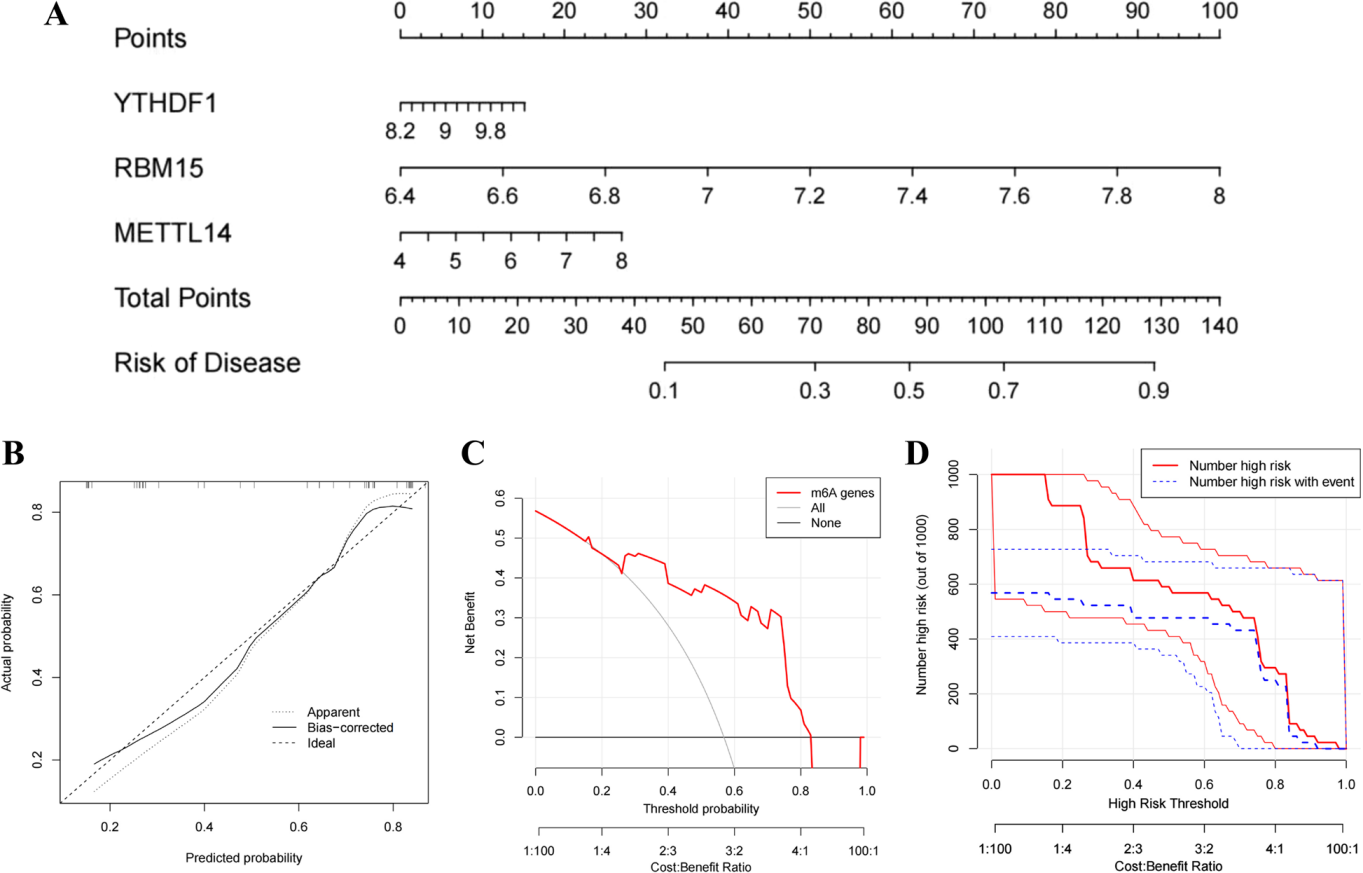
Establishment of a nomogram model. **A** Establishment of a nomogram based on the three selected m6A regulators, YTHDF1, RBM15, and METTL14. **B** Calibration curves of the nomogram indicating predictive robustness of this nomogram. **C** DCA curve revealing the high accuracy and patients’ benefit of the decisions based on this nomogram. **D** Clinical impact curve of the nomogram

Three recommended m6A regulators with a gene importance score above 2 were selected to establish a nomogram for evaluating the occurrence risk of PCOS by the "rms" package of R (Fig. 4A). Based on the multi-factor regression analysis, the nomogram was built to integrate involved genes, score them by scaled line segments, and obtain the total points to predict the occurrence risk of PCOS. The expression levels of these selected genes were positively correlated with the risk score in the nomogram, indicating that they may be risk factors for PCOS patients. The nomogram was consistent with the above analysis based on the expression differences of m6A regulators in control and PCOS patients. The calibration curve displayed that the apparent line, the bias-corrected line, and the ideal line shared a close range and the same tendency, which indicated the superior predictive accuracy of the nomogram (Fig. 4B). The DCA curve demonstrated that the red line developed by the m6A regulator was always at the top of and away from the gray line, which stood for the high accuracy of this nomogram and patients' benefit from the decisions based on this nomogram (Fig. 4C). In addition, the clinical impact curve also proved the nomogram's high benefit and superior predictive performance (Fig. 4D).

### Analysis of subtypes based on m6A regulators

**Fig. 5:**
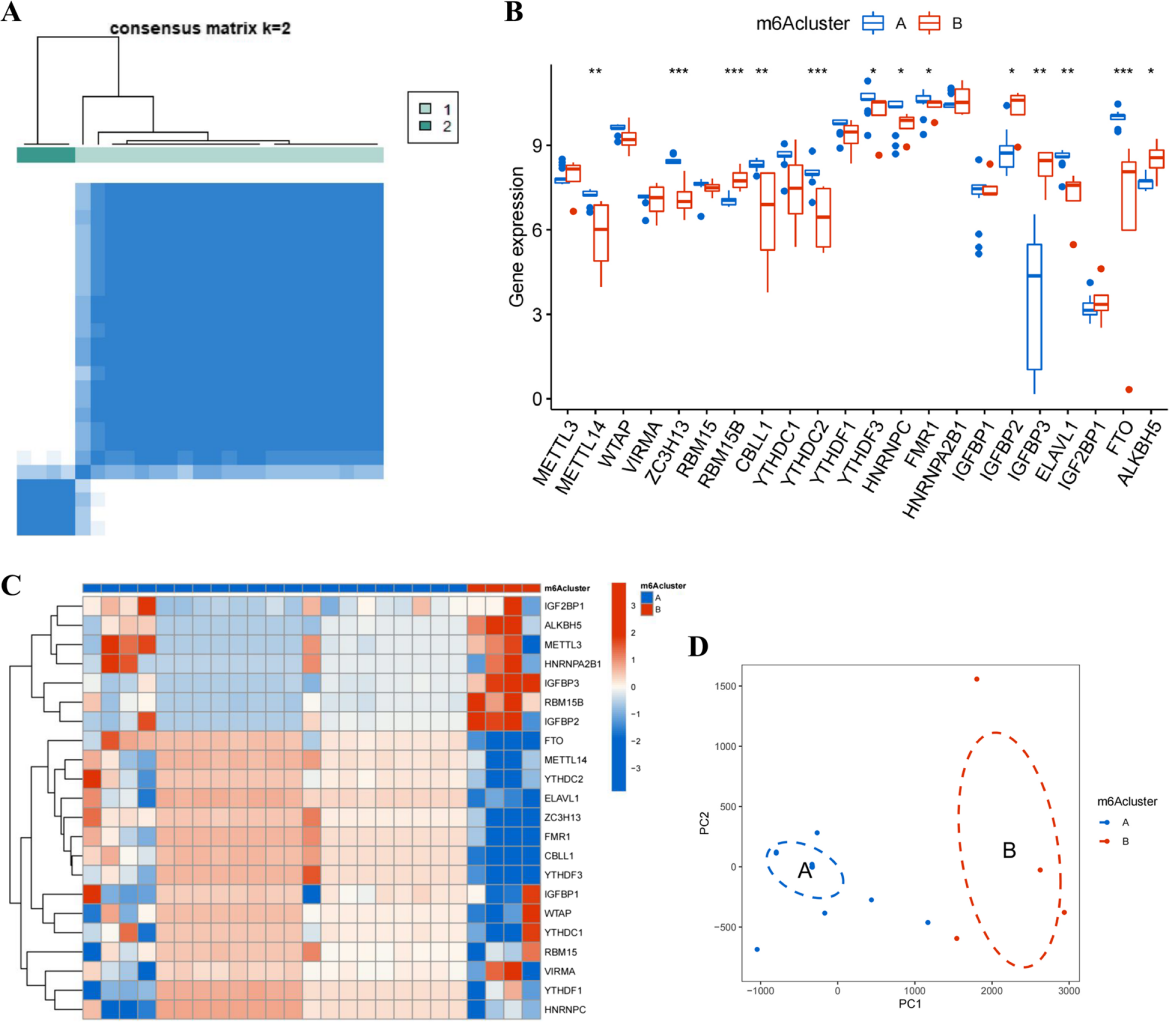
Consensus clustering of 22 DEGs of m6A regulators. **A** Consensus matrix for k = 2. **B** Histogram of expressions of 22 DEGs in m6A regulators between two m6A clusters A and cluster B. **C** Heatmap of expressions of 22 DEGs in m6A regulators between two m6A clusters. **D** Principal component analysis of m6A clusters. *p < 0.05, **p < 0.01, and ***p < 0.001

Based on the 22 differentially expressed m6A regulators, the consensus clustering algorithm was applied to identify different subtypes of PCOS patients. They were well categorized into two clusters when the cluster variable was 2 (Fig. 5A). The expressions of m6A regulators were compared in m6A cluster A and m6A cluster B. The expressions of METTL14, ZC3H13, CBLL1, YTHDC2, YTHDF3, HNRNPC, FMR1, ELAVL1, and FTO presented increased values in cluster A compared to those in cluster B, while the opposite performance was observed in RBM15B, IGFBP2, IGFBP3, and ALKBH5. Furthermore, METTL3, WTAP, VIRMA, RBM15, YTHDC1, YTHDF1, HNRNPA2B1, IGFBP1, and IGF2BP1 showed no significant differences between clusters (Fig. 5B, C). According to the PCA, we could distinctly differentiate the two m6A clusters (Fig. 5D).

### Evaluation of the m6A related gene signature

**Fig. 6:**
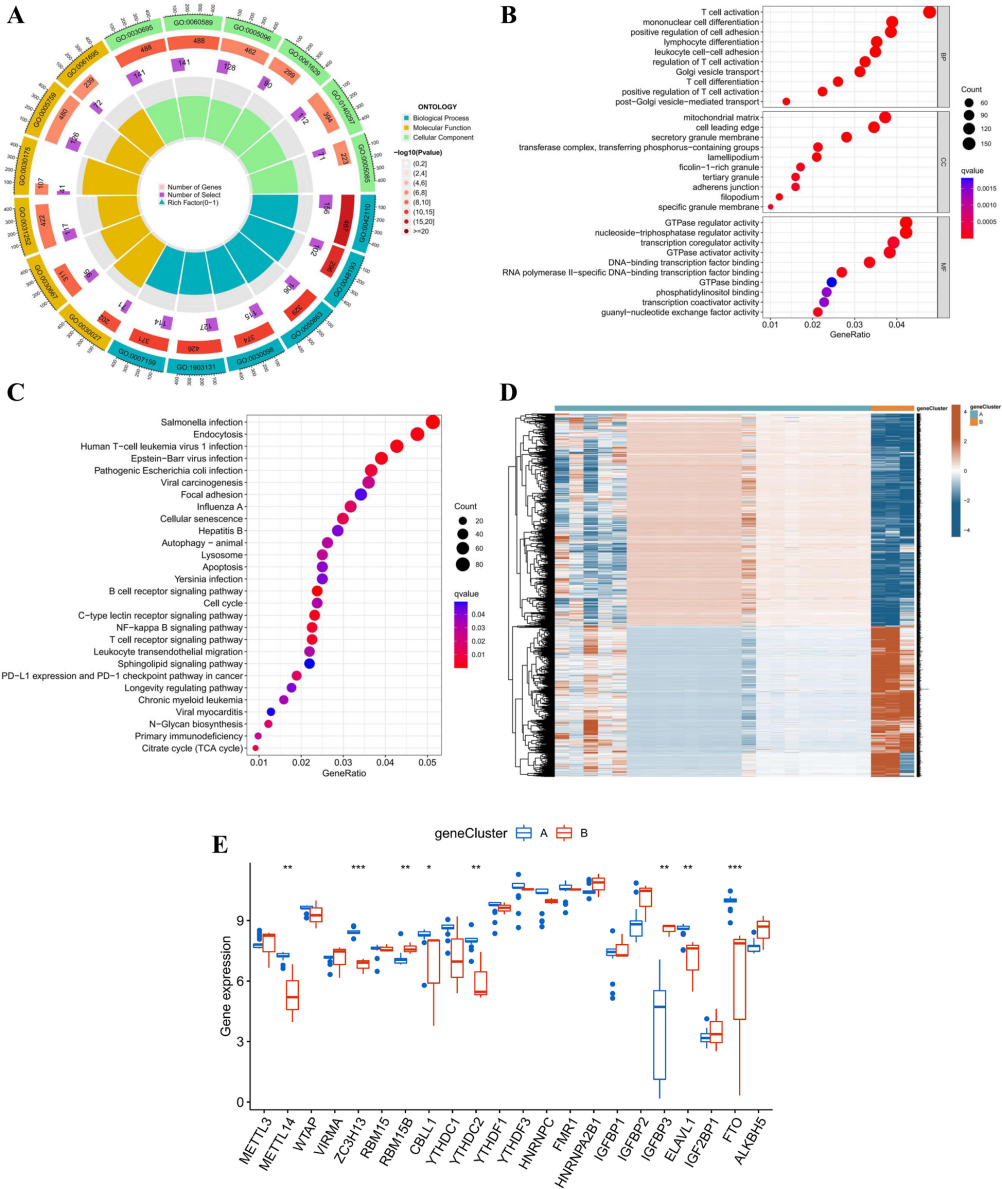
Analysis of the m6A-related DEGs in PCOS. **A** Circle plot of the GO analysis revealing the potential gene functions of the m6A-related DEGs on the occurrence and development of PCOS. **B** Bubble plot of the GO analysis. **C** KEGG analysis of the m6A-related DEGs. **D** Expression heatmap of the m6A-related DEGs in gene cluster A and gene cluster B. **E** Differential expression analysis of the 22 identified m6A regulators in the two gene clusters. *p < 0.05, **p < 0.01, and ***p < 0.001

To attest to the m6A subtypes, the DEGs were identified between the two m6A clusters with an adjusted *p* < 0.01 and a |logFC|≥ 1.5, which were termed the m6A-related DEGs. The GO and KEGG enrichment analyses were performed to explore the potential role of m6A-related DEGs in PCOS. The GO enrichment analysis revealed that the DEGs were especially affluent in immune-related biological processes (Fig. 6A, B). In KEGG enrichment analysis, DEGs were particularly abundant in a variety of infection-related pathways (Fig. 6C). The consensus clustering algorithm was employed to categorize the PCOS patients into two distinct gene subgroups based on these m6A-related DEGs. The two genomic subtypes were in accordance with m6A subtypes, and Fig. 6D displayed the differential expression of these m6A-related DEGs in two gene clusters. The results of differential expressions of m6A regulators between the two gene clusters were also similar to those between the m6A clusters (Fig. 6E).

### Evaluation of the immune cells infiltration patterns

**Fig. 7:**
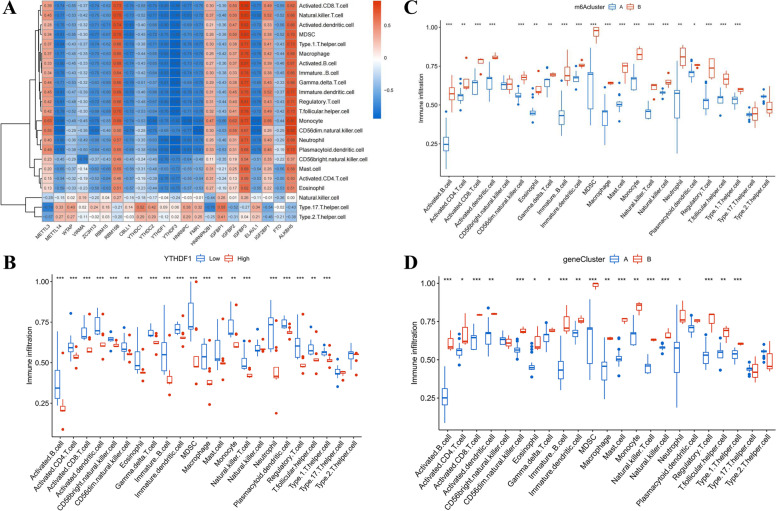
Immune infiltration analysis. **A** Correlation heatmap between infiltrating immune cells and the 22 DEGs of m6A regulators. **B** Differences in the abundance of immune infiltration between high and low YTHDF1 expression levels. **C** Differential expressions of immune cell infiltration between m6A cluster A and m6A cluster B. **D** Differential expressions of immune cell infiltration between gene cluster A and gene cluster B. *p < 0.05, **p < 0.01, and ***p < 0.001

The ssGSEA was conducted to assess the enrichment of immune cells in PCOS specimens and to demonstrate the relationship between the m6A regulators and immune cells (Fig. 7A). Prominent associations were observed between YTHDF1 and various immune cells. Therefore, further investigation was developed into the enrichment of immune cells in PCOS patients with high or low YTHDF1 (Fig. 7B). The boxplot demonstrated that patients with low YTHDF1 expression had obviously enriched immune cells. Ultimately, the differential immune cell enrichment between the m6A clusters was conducted to show the different immune responses of patients in different subtypes. The findings demonstrated that cluster B displayed higher infiltrating levels of immune cells than cluster A, particularly MDSC, activated B cells, and activated CD8 T cells (Fig. 7C), which indicated that patients in m6A cluster B might have a positive immune response for PCOS. The immune infiltration pattern of two gene clusters was in accordance with the pattern of m6A subtypes (Fig. 7D).

### Relationship between m6A and gene subtypes with cytokines

**Fig. 8:**
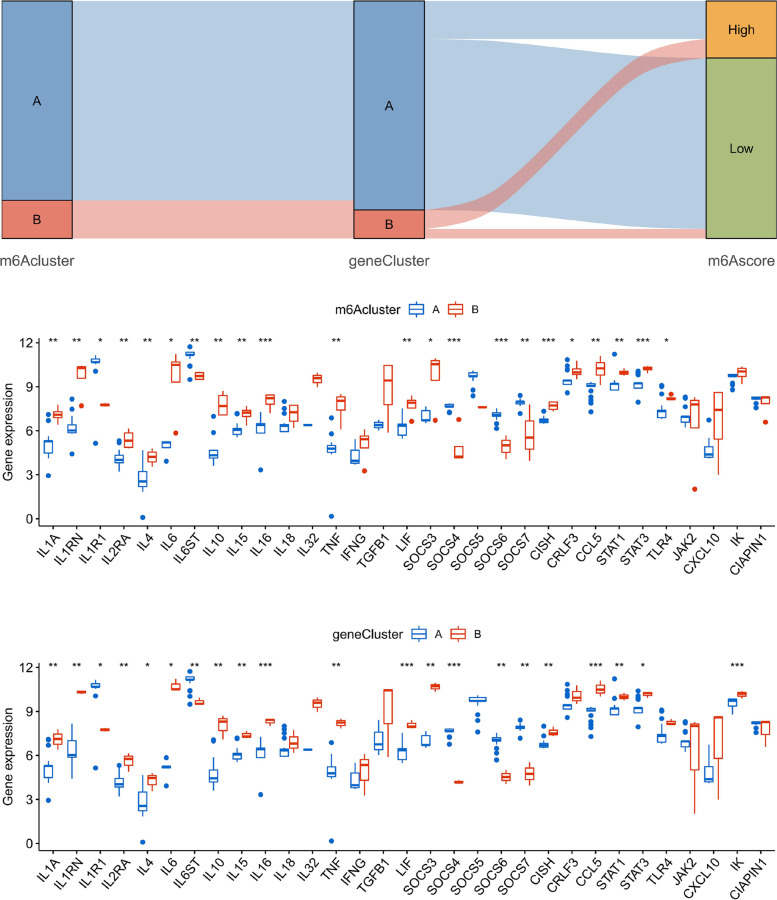
Relationships of m6A subtypes and m6A-related gene subtypes. **A** Sankey diagram of the m6A subtypes, gene subtypes, and m6A scores. **B** Differential expression analysis of cytokines between m6A cluster A and cluster B. **C** Differential expression analysis of cytokines between m6A-related gene clusters. *p < 0.05, **p < 0.01, and ***p < 0.001

In the pathophysiological mechanism of PCOS, pro-inflammatory cytokines play an essential role and lead to inflammation and poor oocyte quality [4]. To further confirm the close correlation between the m6A subtypes and PCOS, a comprehensive exploration of the association between m6A subtypes or m6A-related gene subtypes and cytokines was conducted. As the results displayed, the cytokines presented significant discrepancies in both m6A clusters and gene clusters (Fig. 8B, C). Remarkably, IL1A, IL1RN, IL6, and TNF were overexpressed in m6Acluster B and genecluster B compared to m6Acluster A and genecluster A, which was consistent with existing reports [20–22]. These findings validated the close correlation between m6Acluster B or genecluster B in PCOS.

## Discussion

PCOS is a multifactorial disease caused by genetic, endogenous, endocrine regulation, metabolic disorders, and environmental factors, and the treatment strategies for PCOS involve a combination of lifestyle optimization, medical interventions, which include metformin, hormonal contraceptives, antiandrogens, and so on, and if needed, infertility management [1, 18, 23]. But the diagnosis and following management are often complex and unsatisfactory, highlighting the need for further investigation of the pathogenesis of PCOS. Existing researches have shown that m6A regulators play a role in the biological process of PCOS [18, 24]. As most studies focus on a single m6A regulator, the overall roles of multiple m6A regulators in PCOS are not comprehensively recognized. Identifying the m6A modification patterns in PCOS will contribute to enhancing our understanding of the pathogenesis of the disease and exploring new therapeutic strategies.

**Fig. 9:**
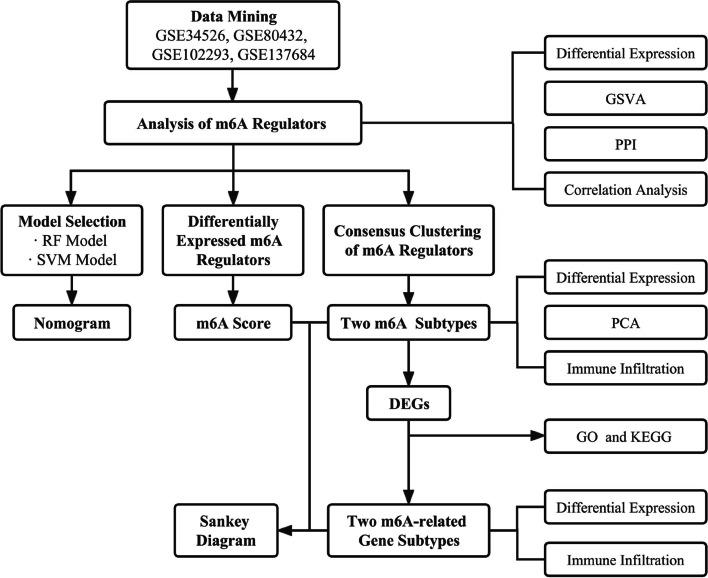
The flow chart of this study

In this study, we comprehensively explored the m6A modification characters in PCOS patients, and the workflow of this study is demonstrated in Fig. 9. The expression profile data of 4 datasets were integrated and analyzed, and the expression levels of m6A regulators were obviously overexpressed in PCOS patients compared to healthy controls, which indicated that m6A modification might be closely related to the development of PCOS. Previous studies have demonstrated the m6A modification levels were increased and the m6A profile was altered in the luteinized GCs of PCOS patients. However, the knockdown analysis of METTL14 proved the absence of METTL14 / m6A / FOXO3 regulation in the luteinized GCs of PCOS patients. But the cell-specific effects of m6A modification are worth investigating and looking for other targets [24]. The elevated expression of FTO may conduce to the dysfunction of GCs by upregulating FLOT2 in PCOS [25]. The expression of IGFBP2 has been reduced in PCOS patients [26] and the levels of IGFBPs are involved in the selection of the dominant follicle [27, 28]. The PPI network was created by STRING to show the relationships between the differentially expressed m6A regulators and to discover hub genes. However, the identified hub genes, METTL3, YTHDF1, and YTHDF3, were obtained from protein interaction relationships of the m6A regulators, which indicated the internal connections of the regulators, and further exploration of the roles of m6A regulators in PCOS required a more comprehensive analysis of the expression levels of m6A regulators.

As a result, RF and SVM models were constructed and compared based on the DEGs of m6A regulators to select a proper model to identify optimal regulators from the DEGs of m6A regulators for predicting the occurrence risk of PCOS. In addition, the RF model has become one of the most popular machine learning algorithms for its high accuracy, favorable robustness, and convenience. Based on the RF model's importance of the m6A regulators, three optimal m6A regulators, YTHDF1, RBM15, and METTL14, were selected to establish a nomogram by multivariate analysis rather than univariate analysis due to the possibility to ignore the multivariate association in the m6A regulators. However, the model could not integrate more disease features of PCOS yet due to the absence of sufficient clinical information and other features of PCOS in the GEO public database. Then, the calibration curve, the DCA curve, and the clinical impact curve further validated this nomogram model and demonstrated that PCOS patients may benefit from the decisions based on this nomogram. In this nomogram, YTHDF1, RBM15, and METTL14 were positively correlated with the risk for PCOS. Though there are few pieces of research on the correlation between three selected m6A regulators and PCOS, previous studies have demonstrated that the three optimal m6A regulators play an essential role in self-renewal and development of female germ stem cells, ovulation, placental insufficiency, and female reproductive system neoplasms such as cervical and endometrial cancer [29–34]. The functions of three selected m6A regulators and this nomogram in PCOS need to be confirmed in further research.

Furthermore, we identified two m6A-related subtypes in PCOS by consensus clustering, named m6A cluster A and m6A cluster B, and obtained m6A-related DEGs between two m6A clusters for identifying gene clusters. The expressions of m6A regulators in m6A cluster A and gene cluster A were significantly higher than those in m6A cluster B and gene cluster B, indicating that the distinction between clusters A and B is essential and warrants additional study. Studies have stated that hormone abnormalities in PCOS can overstimulate the immune system, and concurrently, inflammatory cell infiltration aggravates ovarian dysfunction and metabolic disorders [35, 36]. High infiltrating abundances of macrophages [37], neutrophils, activated dendritic cells [38], and eosinophils [39, 40] were observed in PCOS. Since chronic low-level inflammation plays a critical role in PCOS [41], we investigated and compared the immune infiltration and cytokine levels in different groups. The m6A cluster B presented higher immune cell infiltration behaviors consistent with existing researches, and a similar infiltration trend marked in the genomic subtypes. Additionally, the cytokine levels in m6A cluster B or gene cluster B were significantly higher than those in clusters A, consistent with existing studies [41–43] especially in the up-regulation of IL1A [20], IL1RN [21] and IL6 [22]. Therefore, these results indicated that m6A cluster B and gene cluster B with higher infiltration of immune cells could present more typical characteristics of PCOS, which manifested a higher risk and an inferior clinical performance. Consistent with the findings, PCOS patients in m6A cluster B or gene cluster B obtained higher m6A scores than clusters A.

Currently, the clinical importance and underlying mechanisms of m6A modification in the etiology of PCOS remain unknown, and our findings can help to better understand the roles of m6A involved in PCOS pathogenesis. Additionally, the development of precise gene-targeted therapy [44] possibly provides a new direction for m6A-targeted strategies specifically for PCOS patients. Nonetheless, there are several limitations in the present research. The relatively small number of PCOS patients included in the datasets and the absence of clinical information result in inadequate support to establish a more comprehensive assessment of the specific relationship between m6A regulators and PCOS. The findings are preliminary, and future collaborations on basic, clinical, and epidemiologic research will be critical in determining the roles of m6A in the occurrence and progression of PCOS.

## Conclusion

In conclusion, the m6A regulators might play a role in the pathogenesis of PCOS. We characterized the PCOS with m6A regulators pattern and developed two different m6A subtypes of PCOS, one of which (clusterB) showed higher levels of immune infiltration and cytokines. This study will help clarify the overall impact of m6A modification patterns and related immune infiltration on PCOS. In addition, the developing precise therapy targeting m6A regulators is expected to exert a therapeutic effect on PCOS and the ensuing infertility. Further exploration of the roles of m6A modification in PCOS will help to investigate the pathogenesis and potential therapeutic method of PCOS.

## Data Availability

The data that support the findings of this study are available from the corresponding author upon reasonable request.

## Abbreviations

PCOS: Polycystic ovary syndrome
m6A: N6-methyladenosine
DEG: Differentially expressed gene
HA: Hyperandrogenemia
IR: Insulin resistance
AGE: Advanced glycation end product
GEO: Expression Omnibus
RNA-seq: RNA sequencing
Limma: Linear Models for Microarray Data
PPI: Protein–protein interaction
GSVA: Gene set variation analysis
RF: Random forest
SVM: Support vector machine
ROC: Receiver operating characteristic
DCA: Decision curve analysis
PCA: Principal component analysis
ssGSEA: Single sample gene set enrichment analysis
GC: Granulosa cell
